# An anthropomorphic spine phantom for proton beam approval in NCI‐funded trials

**DOI:** 10.1120/jacmp.v15i3.4742

**Published:** 2014-05-08

**Authors:** Jongmin Cho, Paige A. Summers, Geoffrey S. Ibbott

**Affiliations:** ^1^ Graduate School of Biomedical Sciences at Houston The University of Texas Houston TX; ^2^ Department of Radiation Physics The University of Texas M.D. Anderson Cancer Center Houston TX USA

**Keywords:** proton approval, anthropomorphic spine phantom, RPC, spine matched field, cooperative groups

## Abstract

As part of the approval process for the use of scattered or uniform scanning proton therapy in National Cancer Institute (NCI)‐sponsored clinical trials, the Radiological Physics Center (RPC) mandates irradiation of two RPC anthropomorphic proton phantoms (prostate and spine). The RPC evaluates these irradiations to ensure that they agree with the institutions' treatment plans within criteria of the NCI‐funded cooperative study groups. The purpose of this study was to evaluate the use of an anthropomorphic spine phantom for proton matched‐field irradiation, and to assess its use as a credentialing tool for proton therapy beams. We used an anthropomorphic spine phantom made of human vertebral bodies embedded in a tissue substitute material called Muscle Substitute/Solid Rigid Number 4 (MS/SR4) comprising three sections: a posterior section containing the posterior surface and the spinous processes, and left and right (L/R) sections containing the vertebral bodies and the transverse processes. After feasibility studies at three institutions, the phantom, containing two thermoluminescent dosimeters (TLDs) for absolute dose measurements and two sheets of radiochromic film for relative dosimetry, was shipped consecutively to eight proton therapy centers participating in the approval study. At each center, the phantom was placed in a supine or prone position (according to the institution's spine treatment protocol) and imaged with computed tomography (CT). The images then were used with the institution's treatment planning system (TPS) to generate two matched fields, and the phantom was irradiated accordingly. The irradiated phantom was shipped to the RPC for analysis, and the measured values were compared with the institution's TPS dose and profiles using criteria of ± 7% for dose agreement and 5 mm for profile distance to agreement. All proton centers passed the dose criterion with a mean agreement of 3% (maximum observed agreement, 7%). One center failed the profile distance‐to‐agreement criterion on its initial irradiation, but its second irradiation passed the criterion. Another center failed the profile distance‐to‐agreement criterion, but no repeat irradiation was performed. Thus, seven of the eight institutions passed the film profile distance‐to‐agreement criterion with a mean agreement of 1.2 mm (maximum observed agreement 5 mm). We conclude that an anthropomorphic spine phantom using TLD and radiochromic film adequately verified dose delivery and field placement for matched‐field treatments.

PACS number: 87.55.‐x, 87.55.N‐

## I. INTRODUCTION

Proton therapy is increasingly popular and accessible as one of the most effective forms of cancer treatment. Currently, 11 proton therapy centers operate in the United States, and 13 new centers are in development. As more proton therapy centers become operational, it is desirable to ensure that each center delivers clinically comparable treatment, such as consistent dose. The Radiation Therapy Oncology Group and the Children's Oncology Group, both funded by the National Cancer Institute (NCI), have created proton radiation therapy protocols for clinical trials. Owing to the need to enroll and manage patients with proton therapy in a consistent manner in these NCI‐funded cooperative group clinical trials, the NCI requested that the Radiological Physics Center (RPC) develop a comprehensive approval process. Proton therapy centers wishing to participate in NCI‐sponsored clinical trials can apply for approval by completing the proton facility questionnaire, participating in the RPC's annual remote audit program, submitting electronic treatment planning data, independently irradiating the RPC's baseline proton phantoms with consistent results according to the RPC criteria, and finally, passing an RPC dosimetry review visit. Details of the procedures can be found in the May 2013 issue of the RPC newsletter.^(1)^

Regarding the fourth requirement (irradiating the RPC's baseline proton phantoms), irradiation of two RPC anthropomorphic phantoms (prostate and spine) is required for approval of scattered or uniform scanning proton beams. The prostate phantom has been described previously^(2)^ although for proton irradiation, small changes were made to angle the radiochromic films slightly relative to the central axes of the proton beams. The purpose of the prostate phantom is to evaluate an institution's ability to correctly plan and deliver a prostate treatment with protons, including properly making heterogeneity corrections. The spine phantom was constructed to evaluate the ability of the participating institutions to correctly and accurately match proton beams at depth within a patient, as might be necessary to treat the craniospinal axis in a patient.

## II. MATERIALS AND METHODS

### A. Phantom design

In 1991, G.S. Dominiak developed a rectangular spine phantom to evaluate the craniospinal electron irradiation technique that was used by The University of Texas M. D. Anderson Cancer Center at that time.^(3)^ To construct the phantom, human vertebral bodies were suspended in a container and tissue substitute material identified as “muscle substitute, solid rigid number 4” (MS/SR4)^4^, ^5^ was poured in around them. The MS/SR4 gradually hardened, resulting in a solid phantom with human vertebral bodies (T9 through L4) embedded within. The phantom was cut into three pieces. The posterior section contained the posterior surface and the spinous processes. The left and right [L/R] sections contained the vertebral bodies and the transverse processes. To measure the treatment beam dose profile, film sheets were placed between the different sections.^(3)^

**Figure 1:**
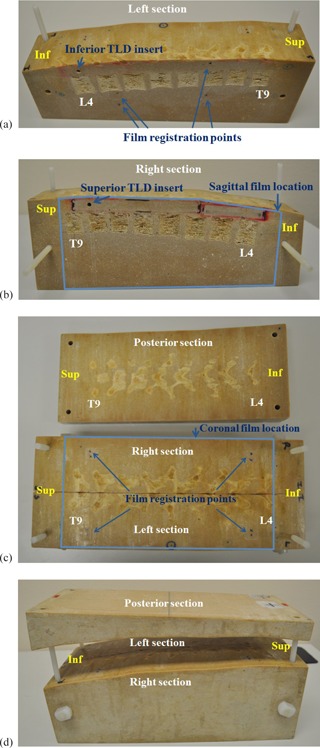
Anthropomorphic spine phantom evaluated for use in the Radiological Physics Center's proton approval process for National Cancer Institute‐funded clinical trials: (a) left section of the phantom; (b) right section of the phantom; (c) the combined L/R section of the phantom (bottom) and the posterior section of the phantom (top); (d) configuration of the posterior section combined with the combined L/R section. When the posterior section is collapsed, the phantom is completely combined.

This phantom was subsequently tested for use in evaluating craniospinal proton irradiation at the UT M. D. Anderson Cancer Center Proton Therapy Center (Houston, TX). By placing one sagittal film between the L/R sections and one coronal film between the posterior section and the combined L/R sections, the accuracy of proton matched fields was tested. The sagittal film was parallel to the field central axes, but the coronal film lay along a curved slope of the vertebrae where the phantom was cut. Figs. 1(a) and 1(b) show the left and right sections of the spine phantom (prone position). The cross section of vertebral bodies is shown: T9 stands for 9th thoracic vertebra, and L4 stands for 4th lumbar vertebra of the spine. The medial surface of the left section of the phantom has four permanently marked film registration points whose positions, relative to phantom edges, are known (Fig. 1(a)). A sagittal film was securely taped on the left section of the phantom, marked with four film registration points using a permanent marker and sandwiched with the right section of the phantom. Figure 1(b) shows the location of the sagittal film relative to the phantom. Figure 1(c) shows the combined L/R section of the phantom at the bottom of the picture and the posterior section of the phantom at the top of the picture. The posterior surface of the combined L/R section of the phantom also has four film registration points (Fig. 1(c)). A coronal film was placed and securely taped on top of this plane and four film registration points were marked on the film using a permanent marker. The combined L/R section of the phantom and the posterior section of the phantom were tightly screwed together (Fig. 1(d)) to prevent film (both sagittal and coronal) movements.

### B. Feasibility studies

We chose the spine phantom as a baseline proton phantom for the NCI approval process to evaluate the accuracy with which clinical trials participants delivered complex spine matched‐field treatments. By evaluating each proton therapy center's matched fields, the RPC can evaluate the accuracy of its treatment delivery system as a whole because the treatment steps include computed tomography (CT) simulation, treatment planning, digital imaging (DI), and treatment beam delivery. The human vertebral bodies in the phantom were essential for evaluating each proton therapy center with a realistic spine matched‐field treatment scenario. The first feasibility study was performed at M.D. Anderson. The phantom was imaged, and treatment was planned and delivered as if the phantom were an actual patient, using M.D. Anderson's craniospinal irradiation (CSI) proton protocol.

**Figure 2:**
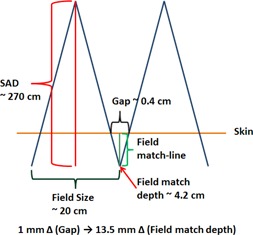
Feasibility study treatment setups. According to the formula of similar triangles, 1 mm uncertainty in gap causes 13.5 mm uncertainty in field match depth (diagram not to scale).

First, two sheets of radiochromic film (GAFCHROMIC EBT2, Ashland Inc., Covington, KY) were inserted into the sagittal and coronal planes of the phantom. Each film was cut to cover almost the entirety of each plane and marked with four film registration points whose distances from the phantom edges are known (Figs. 1(a), 1(b), and 1(c)). These registration points were used to coregister film positions relative to the treatment plan CT images in which dose calculation was performed. The phantom with films inserted was then placed on a CT couch in the prone position for CT simulation. The phantom was scanned using a representative CSI protocol from M.D. Anderson (2.5 mm thick slices, 120 kVp, and 350 mA). A passive proton beam plan was generated using a treatment planning system (TPS) (Eclipse; Varian Medical Systems, Palo Alto, CA). In this plan, two matched fields were generated to cover the spinal cord of the phantom, and the field separation was calculated to match the field at a depth of 4.2 cm, which corresponds to the anterior part of the spinal cord adjacent to the L1 vertebral body. Figure 2 shows a simplified treatment configuration. It is important to note that, according to the principle of similar triangles, a small error in gap thickness (1 mm) creates a significant change (13.5 mm) in field match depth.

For the proton exposure, the Gantry 2 passively scattered beam of the M.D. Anderson Proton Therapy Center (Hitachi PROBEAT, Chiba, Japan) was used. The phantom was positioned parallel to and on top of the treatment couch, using the patient setup lasers. Two digital kV images (anterior/posterior [A/P] and lateral [LAT]) were taken, and the phantom was aligned to the superior field isocenter by comparing digitally reconstructed radiographs (DRRs) with the kV digital images. The couch was adjusted accordingly, and the superior field was irradiated. The couch was then moved longitudinally to the inferior field isocenter, as prescribed by the treatment plan. Two orthogonal kV digital images of the phantom were taken and compared with the corresponding DRRs for the inferior field. According to the kV digital image setup alignment suggestions, the couch was shifted an additional 1 mm laterally and 1.5 mm longitudinally to the inferior treatment isocenter. After the shift, positioning was confirmed with another pair of orthogonal kV digital images, and then the inferior field was irradiated. A unique set of apertures and compensators was used for each field. A dose of 6 Gy was delivered at the isocenter of each field.

Additional feasibility studies were performed at two other proton therapy centers. Under the instruction of RPC personnel, the phantom was simulated with CT, and treatment was planned and delivered using each institution's CSI treatment protocol. Although RPC personnel were present during irradiation, each institution's personnel performed the entire procedures independently so that the institution could use the feasibility studies, if successful, in the NCI approval process. Consequently, these institutions are not identified in this report.

Exposed film was scanned using a 633 nm laser densitometer (Photoelectron Corp, Lexington, MA) with a scanning resolution of 0.1 mm. Details of the film digitization method used in this study were described by Molineu et al.^(6)^ The dose distributions obtained from film measurement were compared with the institution‐provided dose distribution from their TPS calculation to determine the field agreement in both the sagittal and coronal planes. On the sagittal film, dose profiles along the superior‐inferior (S/I) direction were obtained at three different depths (2.7, 3, and 4 cm), and in the coronal plane, a single dose profile along the S/I direction was obtained. Using those profiles, the matchline location agreement (within 5 mm), which is defined as match‐line distance to agreement, was used as the criteria for approval. A Computational Environment for Radiotherapy Research (CERR) software platform^(7)^ was used for film and treatment plan coregistration, as well as for dose profile analysis. The spatial resolution and accuracy of the film and digitizing system (densitometer) was 1 mm.

### C. Proton center approved studies

After the feasibility studies were completed at M.D. Anderson and the two other institutions, the phantom was modified to accommodate two thermoluminescent dosimeter (TLD) capsules in superior and inferior locations for absolute dose measurement (see Figs. 1(a) and 1(b) for superior and inferior TLD locations). The TLD locations were chosen to be in low‐dose gradient regions but near the edges of the phantom to avoid interfering with film measurements. Additionally, the dose profile analysis method was modified to overcome the limitation of the analysis method discovered from the feasibility studies. Instead of obtaining only S/I profiles on both films, one or two S/I profiles and an A/P profile were obtained on the sagittal film, and two L/R profiles were obtained on the coronal film. The A/P profile was obtained in either the superior or inferior field, but about 1 to 2 cm away from the field matchline where the dose gradient was small. L/R profiles were obtained across superior and inferior matched fields about 1 to 2 cm symmetrically away from the field matchline in small dose gradient areas.

The phantom, inserted with new TLDs and unexposed film, was then shipped to eight participating proton centers (including M.D. Anderson Proton Therapy Center) for NCI approval. Two previous proton therapy centers participated in the feasibility studies were not included in this proton approval studies since their feasibility study results, supplemented by independent dose measurement results, were determined to be acceptable for NCI approval. The film was cut to the edges of the phantom, taped, and clamped to prevent film movement during shipment. Upon arrival, each center imaged the phantom and two matched fields were generated using each center's TPS and irradiated according to each center's proton CSI (or spine matched field) treatment protocol. Instructions were given to handle and treat the phantom as if a real patient. All proton therapy centers' treatment protocols were comparable to that of our feasibility study at the M.D. Anderson Proton Therapy Center. Exposed film and TLDs were shipped back to the RPC for film digitization, TLD reading, and analysis. Each center's calculated dose and treatment plan were delivered through the file‐sharing program of the Image‐Guided Therapy Center (ITC). After the completion of each approval study, a comprehensive report indicating NCI approval was generated and sent to the institution.

### D. Dosimeters

Radiochromic film is approximately tissue‐equivalent, insensitive to light, has a relatively small angular dependence, and is widely used as a relative dosimeter to measure beam profiles owing to its excellent spatial resolution.^8^, ^9^ Absolute dose measurement was obtained using approximately 40 mg TLD‐100 powder in the TLD capsules, which were inserted in the phantom for irradiation. The capsules were made of high‐impact polystyrene and were cylindrical (5 mm in diameter×5 mm long), with 1 mm thick walls. Irradiated TLD‐100 powder was moved to a TLD readout system (Harshaw TLD Model 4500; Thermo Fisher Scientific, Waltham, MA) to measure the light output, from which the absolute dose was calculated after accounting for factors such as the mass of the powder, fading, and energy dependence. The TLD system has dosimetric accuracy and precision of ±4% and ±3%, respectively, and can detect dosimetric errors equal to or greater than ±5%.^10^, ^11^ (See Molineu et al.^(6)^ for details of the dosimeters and analysis methods used in this research.) Throughout the feasibility and approval studies, a ±7% agreement criterion was used between the TLD‐measured dose and the TPS‐calculated dose, a 5 mm distance‐to‐agreement criterion was used for both profile and matchline distance to agreement. These criteria are consistent with current RPC practices.

## III. RESULTS & DISCUSSION

### A. Feasibility studies

**Figure 3:**
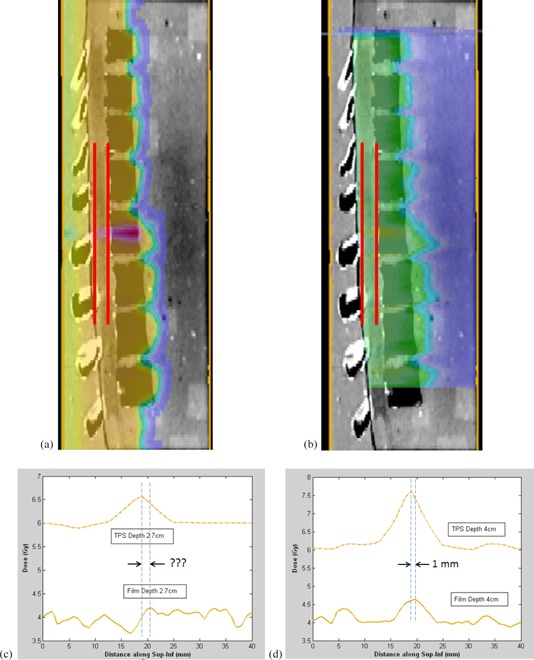
Feasibility study — sagittal plane: (a) proton isodose distribution in the sagittal plane from the treatment planning system (TPS) calculation overlaid on the computed tomography (CT) image; (b) sagittal film‐measured isodose distribution overlaid on the CT image where the two red lines represent the two locations where superior/inferior (S/I) profiles were obtained; (c) dose profile comparison between the TPS calculation and the film measurement across the S/I profile (left red line) at a depth of 2.7 cm; (d) comparison at a depth of 4 cm (right red line).

**Figure 4:**
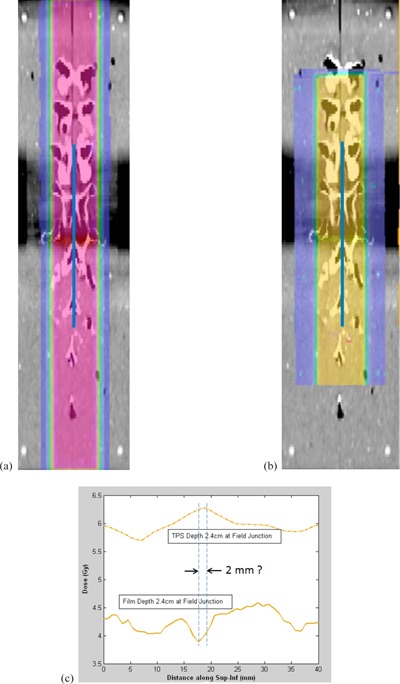
Feasibility study — coronal plane: (a) proton isodose distribution from the TPS calculation in the coronal plane overlaid on the CT image; (b) coronal film‐measured isodose distribution overlaid on the CT image in which the blue line represents the location where the S/I profile was obtained; (c) proton dose profile comparison between the TPS calculation and the film measurement across the S/I profile (blue line).

Feasibility studies performed at three proton therapy centers, including M.D. Anderson, demonstrated that good agreement (better than 4 mm) can be achieved between the film measurement of the location of the beam matchline and the TPS profile. The dosimetric tendencies (hot spot or cold spot) seem to agree between the TPS profile and the film, though this agreement was not apparent for some profiles obtained at various depths. For example, Figs. 3 and 4 show results from the proton feasibility study at one of the centers. Figure 3(a) shows the calculated proton isodose distribution in the sagittal plane overlaid on the CT image. Figure 3(b) shows the corresponding measured isodose distribution from the exposed and digitized film. Red lines on each figure show two locations (depths of 2.7 and 4 cm) where the S/I profiles were obtained. A cold spot (green) at the surface gradually changes to a hot spot (red) as depth increases, as seen in Fig. 3(a). The TPS distribution, which shows the matched field from a depth of 0, clearly shows the field junction (Fig. 3(a)); however, the cold spot of the field junction does not show as clearly for the film since the minimum film depth is 2.4 cm (Fig. 3(b)). Figures 3(c) and 3(d) show the dose profile comparison between the TPS profile and the film along the S/I direction on the sagittal plane at depths of 2.7 and 4 cm, respectively. At 4 cm depth, the TPS profile and the film show clear hot spots and dosimetric similarity at the location of the matchline. However at the shallower depth of 2.7 cm, the matchline is not as evident on the film. Therefore, Figs. 3(d) and 3(c) show good and bad examples of choosing the depth where matchline distance to agreement can be obtained. After comparing distance to agreement of field matched locations at multiple depths where clear matchline can be located, the maximum value was recorded as the institution's sagittal distance to agreement, as shown in Table 1. Note that the absolute dose was not compared here because the radiochromic film was used to compare relative dose profiles, and the TLDs were not yet in place in the phantom for scaling the film.

**Table 1:** Field matchline distance to agreement between film measurement and TPS calculation in feasibility studies using an anthropomorphic spine phantom for the Radiological Physics Center's proton approval process for National Cancer Institute‐funded clinical trials. Dosimetric tendency (hot spots or cold spots) similarity was compared qualitatively

	*Matchline Distance To Agreement*		
*Institution*	*Sagittal*	*Coronal*	*Criterion*
1	4 mm	4 mm	5 mm
2	3 mm	3 mm	5 mm
3	2 mm	2 mm	5 mm

Figure 4(a) shows the calculated proton isodose distribution in the coronal plane overlaid on the CT image. Figure 4(b) shows the corresponding measured isodose distribution from the exposed and digitized film. The blue line represents the location where the S/I profile was obtained. Figure 4(c) shows that the TPS profile appear to show a small hot spot at field junction (matchline); however, the film profile does not show a prominent hot or cold spot. In this case, although debatable, the most prominent peak or valley in the profile (cold spot in this case) was chosen as field junction and compared with the TPS profile for the distance to agreement (2 mm), as well as for matchline dosimetric agreement. No quantitative criterion for dosimetric agreement was established in this research. A more accurate quantitative comparison was not possible because no absolute dose measurement was performed in this feasibility study and, additionally, the film lay in a curved plane that deviated from a true coronal plane and the film lay in uniform dose region which does not show a prominent cold or hot spot. Since the feasibility study showed shortcomings in the quantitative evaluation of the matchline positioning, further phantom analysis for the NCI approval did not include a quantitative criterion for matchline distance to agreement.

Despite imperfections in the analysis method, the results from the feasibility studies from all three centers showed agreement within 4 mm in matchline distance to agreement between the TPS profile and the film (Table 1). Although not included in this report, separate proton dose measurements were performed using both mailed TLDs and a separate phantom irradiation for the two external proton therapy centers participating in this feasibility study. Both institutions passed the dose acceptance criterion and, therefore, were issued NCI approvals.

### B. Proton center approved studies

**Figure 5:**
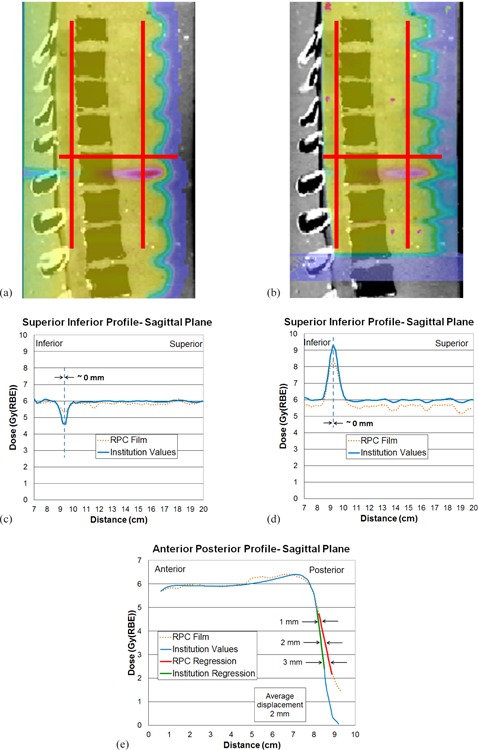
Approval study — sagittal plane: (a) TPS‐calculated proton isodose distribution in the sagittal plane overlaid on the CT image; (b) the sagittal film‐measured isodose distribution overlaid on the CT image where the red lines indicate the region of the anterior/posterior (A/P) and two S/I profile measurements; (c) dose profile comparison between the TPS calculation and the sagittal film measurement in the S/I direction at a depth of 3.9 cm; (d) dose profile comparison between the TPS calculation and the film measurement in the S/I direction at a depth of 9.3 cm; (e) dose profile comparison between the TPS calculation and the film measurement in the A/P direction.

**Figure 6:**
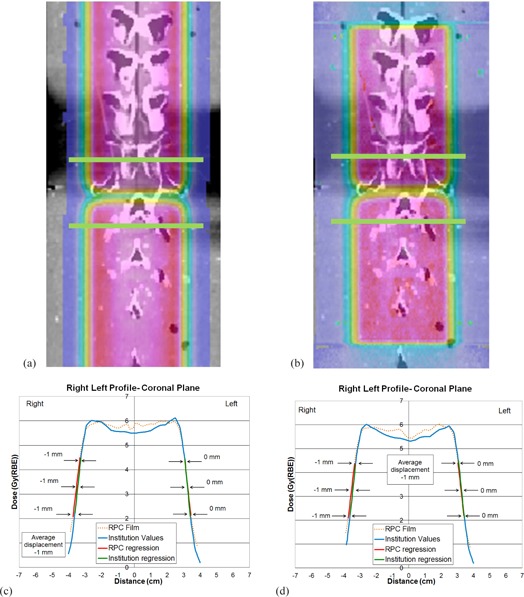
Approval study — coronal plane: (a) TPS‐calculated proton isodose distribution in the coronal plane overlaid on the CT image; (b) coronal film‐measured isodose distribution overlaid on the CT image with green lines indicating the region of the left/right (L/R) profile measurements for the superior and inferior fields; (c) proton dose profile comparison between the TPS calculation and the film measurement across the superior L/R green line; (d) proton dose profile comparison between the TPS calculation and the film measurement across the inferior L/R green line.

The modified spine phantom with two TLD capsules was sent out to eight centers, of which none (except M.D. Anderson Proton Therapy Center) was included in the feasibility study. Table 2 shows the ratio of RPC‐measured TLD dose and the dose calculated by the institution with its TPS at the two TLD locations. Figures 5 and 6 show results from one of the phantom irradiations. Figure 5(a) shows the TPS‐calculated proton isodose distribution in the sagittal plane overlaid on the CT image. Figure 5 (b) shows the corresponding measured isodose distribution from the exposed and digitized film. Three red lines are drawn on this plane — two in the S/I direction (at depths 3.9 and 9.3 cm) and one in the A/P direction. Figure 5(c) shows the dose profile comparison between the TPS calculation and the film measurement in the S/I direction at a depth of 3.9 cm (left red line). Figure 5(d) shows the dose profile comparison in the S/I direction at a depth of 9.3 cm (right red line). Unlike film profiles from Figs. 3 and 4 which show significant differences in baseline doses (in the uniform dose regions) with TPS profiles, film profiles from Figs. 5 and 6 are normalized to the TLD dose measurement and, therefore, show very similar doses compared with TPS profile baseline doses. Figures 5(c) and 5(d) show similar cold and hot spots, respectively, and near 0 distance to agreement between the TPS‐calculated and film‐measured dose profiles. However, not all profiles in the S/I direction showed prominent cold or hot spots, but instead showed uniform field matches; therefore, the matchline distance to agreement was not used as a criterion for NCI proton beam approvals. A single S/I profile was examined in each institution, except for two institutions (one that repeated the irradiation and another where two profiles at two different depths were examined).

**Table 2:** Ratio between RPC‐measured TLD dose and institution‐calculated TPS dose in approval studies

*Institution*	*Superior TLD Dose/Institution TPS Dose*	*Inferior TLD Dose/Institution TPS Dose*	*Criterion*
3	0.98	0.99	0.93−1.07
3 repeat^a^	1.01	0.95	0.93−1.07
4	1.00	1.00	0.93−1.07
5	0.98	0.97	0.93−1.07
6	0.95	0.93	0.93−1.07
7	0.97	0.96	0.93−1.07
8	0.99	0.95	0.93−1.07
9	1.00	0.97	0.93−1.07
10	0.97	0.96	0.93−1.07
Average	0.98	0.96	Mean: 0.97
Standard Dev.	0.02	0.02	

a Testing was repeated at institution 3 after the initial test exceeded the profile distance‐to‐agreement 5 mm criterion. TLD=thermoluminescent dosimeter.

Figure 5(e) shows the dose profile comparison in the A/P direction in the sagittal plane. The difference between the TPS and film profiles was measured at three points in the distal dose falloff region (75%, 55%, and 35% of the maximum dose), and their average was defined as the distance to agreement. Figure 6(a) shows the calculated proton isodose distribution in the coronal plane overlaid on the CT image, and Fig. 6(b) shows the corresponding measured dose distribution from the exposed and digitized film. Two green lines are drawn from left to right on both films near the beam matchline. Along each green line, two dose profiles were created—one from the TPS calculation and another from the film measurement. Figures 6(c) and 6(d) show comparisons of the two profiles at the locations of the superior and inferior green lines, respectively, in Fig. 6(a) and 6(b). The difference between the TPS and film profiles was measured at three points (75%, 55%, and 35% of maximum dose) in each of the penumbra regions. The averages were calculated from both the left and right penumbra regions, and the larger value was taken as the distance to agreement. Values from Figs. 5(e), 6(c), and 6(d) are tabulated in Table 3, which shows the distance‐to‐agreement between the film measurement and the TPS calculation for three profiles: A/P profile taken in the sagittal plane, and L/R profiles taken in the coronal plane within the superior field and within the inferior field. In Tables 2, 3, and 4, the institutions were identified by numbers 3 through 10 since two institutions participated in the feasibility study but not in the approval study. All RPC/institution dose ratios agreed within the criterion of ±7%. Including the institution that repeated the irradiation, all institutions but one passed the 5 mm criterion of the distance‐to‐agreement measurements for beam profiles.

**Table 3:** Profile distance to agreement between film measurement and TPS calculation in approval studies

*Institution*	*Sagittal A/P*	*Coronal L/R Superior*	*Coronal L/R Inferior*	*Criterion*
3	0 mm	3 mm	6 mm	5 mm
3 repeat^a^	−1 mm	2 mm	1 mm	5 mm
4	9 mm	2 mm	7 mm	5 mm
5	−1 mm	1 mm	2 mm	5 mm
6	0 mm	1 mm	2 mm	5 mm
7	2 mm	−1 mm	−1 mm	5 mm
8	2 mm	1 mm	1 mm	5 mm
9	4 mm	1 mm	1 mm	5 mm
10	1 mm	3 mm	5 mm	5 mm
Average	1.8 mm	1.4 mm	2.7 mm	Mean values after excluding institutions 3 & 4: 1.2 mm
Standard Dev.	3.2 mm	1.2 mm	2.7 mm	

a Testing was repeated at institution 3 after the initial test exceeded the profile distance‐to‐agreement 5 mm criterion. TPS=treatment planning system; A/P=anterior/posterior; L/R=left/right; SD=standard deviation.

**Table 4:** Matchline distance to agreement in approval studies

*Institution*	*Matchline Distance To Agreement*	*Criterion*
3	uniform^b^	5 mm
3 repeat^a^	uniform^b^	5 mm
4	2 mm	5 mm
5	3 mm	5 mm
6	3 mm	5 mm
7 profile 1	1 mm	5 mm
7 profile 2^c^	1 mm	5 mm
8	uniform^b^	5 mm
9	4 mm	5 mm
10	2 mm	5 mm
Average	2.3 mm	Data from institutions
Standard Dev.	1.1 mm	3 and 8 not used.

a Testing was repeated at institution 3 after the initial test exceeded the profile distance‐to‐agreement 5 mm criterion.

b Uniform stands for no prominent cold or hot spots observed in either (or both) film or/and treatment planned dose profiles.

c Two profiles were obtained for institution 7.

There are several limitations in this research. Proton matched fields are not a simple sum of two uniform proton fields whose dose increases with depth (from cold spot to hot spot as depth increases, as is the case for the X‐ray matched fields). Instead, beam apertures and compensators are carefully designed during treatment planning to provide as uniform as possible dose distribution over the entire matchline. For this reason, no prominent cold or hot spots (rather uniform dose) were observed either (or both) in TPS and film for institutions 3 and 7. These uniform dose profiles prevent accurate matchline distance‐to‐agreement analysis. Additionally, no quantitative comparison of dosimetric agreement in matchline (hot or cold spot) could be performed. As can be seen from a simplified field match diagram in Fig. 2, small differences in matched‐field gap creates significant changes in field match depth which deemed to cause significant dosimetric changes; however, no quantitative dosimetric analysis was performed at the matchline. Further work should be done to develop a new method for analyzing the spine fields' matchline. Table 4 shows the retrospective analysis of matchline distance to agreement for the proton center approval studies. Although this evaluation was not application for institu3 and 8, most institutions passed a criterion (which was not used for the NCI approval) of 5 mm.

Despite these limitations, the phantom did prove a useful tool in the evaluation of proton dose delivery for dose, depth dose, and lateral profiles. Out of the eight participating centers that irradiated the modified phantom for the NCI approval process, one center did not meet the film distance‐to‐agreement criterion (5 mm) in beam profiles. All participating centers passed the TLD and TPS dose comparison within the criterion (minimum observed deviation, 0%; maximum observed deviation, 7%), as well as the beam profile distance‐to‐agreement criterion (minimum observed distance, 0 mm; maximum observed distance, 4 mm).

## IV. CONCLUSIONS

The feasibility studies showed that the anthropomorphic spine phantom was adequate for the NCI approval process for proton beam irradiation. The human vertebral bodies embedded in the spine phantom provided a realistic patient treatment setting to test spine matched‐field treatments. The spine phantom was adopted as an RPC baseline phantom, and studies were performed with ten participating proton centers. The 7% and 5 mm acceptance criteria have subsequently been adopted by the RPC for the phantom analysis.

## ACKNOWLEDGMENTS

The authors thank all participating proton therapy centers. The authors also thank Ryan Grant, Nadia Hernandez, Richard Amos, Narayan Sahoo, Michael Gillin, Andrea Molineu, Paola Alvarez, Ashley Hollan, Jessica Lowenstein, Richard Wu, and Rebecca Wu in M.D. Anderson Cancer Center's Department of Radiation Physics; M.D. Anderson's Department of Scientific Publications. This work was supported by the Federal Share of program income earned by

Massachusetts General Hospital on C06 CA059267, Proton Therapy Research and Treatment Center, and by grants CA010953 and CA081647 awarded by the National Cancer Institute, United States Department of Health and Human Services. This research is also supported in part by the National Institutes of Health through the M.D. Anderson Cancer Center Support Grant (P30 CA016672).
